# The Average Age of Atrioventricular Block Onset in Middle Eastern Patients with Cardiac Rhythm Devices Adjusted for the Overall Young Population: Insights from a Multicenter International Registry

**DOI:** 10.5334/gh.1321

**Published:** 2024-04-25

**Authors:** Abdulelah H. Alsaeed, Fawziah Al Kandari, Raed Sweidan, Fayez Bokhari, Ahmed Al Fagih, Abdulmohsen Almusaad, Bander Alghamdi, Amir Abdelwahab, Saad AlHasaniah, Ahmed Hersi, Wael Alqarawi

**Affiliations:** 1 Department of Cardiac Sciences, College of Medicine, King Saud University, Riyadh, Saudi Arabia; 2 Chest Diseases Hospital, Kuwait; 3 King Fahad Armed Forces Hospital, Jeddah, Saudi Arabia; 4 Prince Sultan Cardiac Center, Riyadh, Saudi Arabia; 5 King Abdulaziz Medical City -National Guard Health Affairs, Riyadh, Saudi Arabia; 6 King Faisal Specialist Hospital & Research Center, Riyadh, Saudi Arabia; 7 Cairo University Hospital, Cairo, Egypt; 8 King Fahad Military Medical Complex, Dhahran, Saudi Arabia; 9 University of Ottawa Heart Institute, University of Ottawa, Ottawa, Canada

**Keywords:** Atrioventricular block, Average age, Presentation, Middle East, Developing countries, Population characteristics

## Abstract

**Background:**

Previous registries have shown a younger average age at presentation with cardiovascular diseases in the Middle East (ME), but no study has examined atrioventricular block (AVB). Moreover, these comparisons are confounded by younger populations in the ME. We sought to describe the average age at presentation with AVB in ME and quantify the effect of being from ME, adjusted for the overall younger population.

**Methodology:**

This was a retrospective analysis of PANORAMA registries, which collected data on patients who underwent cardiac rhythm device placement worldwide. Countries with a median population age of ≤30 were considered ‘young countries’. Multivariate linear regression was performed to assess the effect of being from ME, adjusted for being from a ‘young country’, on age at presentation with AVB.

**Results:**

The study included 5,259 AVB patients, with 640 (8.2%) from the ME. Mean age at presentation was seven years younger in ME than in other regions (62.9 ± 17.8 vs. 70 ± 14.1, P < 0.001). Being from a ‘young country’ was associated with 5.6 years younger age at presentation (95%CI –6.5––4.6), whereas being from ME was associated with 3.1 years younger age at presentation (95%CI –4.5––1.8), (P < 0.001 for both).

**Conclusion:**

The average age at presentation with AVB in the ME is seven years younger than in other regions. While this is mostly driven by the overall younger population, being from the ME appears to be independently associated with younger age. Determinants of the earlier presentation in ME need to be assessed, and care should be taken when applying international recommendations.

## Introduction

The age at presentation with atrioventricular block (AVB) is essential in assessing the need to rule out secondary causes of AVB. Indeed, consensus documents recommend evaluating patients presenting with AVB for cardiac sarcoidosis if they are 60 or younger, without taking into consideration the population-specific average age at presentation [1]. No data is available pertaining to the average age at presentation with AVB in the Middle East (ME). However, a consistent observation seen in multiple cardiovascular diseases’ (CVD) registries in the ME is the lower average age at presentation compared to their western counterparts [2,3,4,5,6,7,8,9,10,11,12]. For example, the average age at presentation with acute coronary syndrome (ACS), heart failure, and atrial fibrillation in the ME is ≥10 years lower than in western populations [2,3,4,5,6,7,8,9,10,11,12].

These observations, if not cautiously interpreted, might suggest that patients from the ME develop CVD ≥10 years earlier than patients from western countries. However, it is important to note that the average age of the Middle Eastern populations is ≥10 years younger than the west [13]. Indeed, the median ages of the population in Saudi Arabia and Egypt in 2010 were 26 and 24 years, respectively, as opposed to 37 and 40 years in the United States of America and the United Kingdom, respectively [13]. This younger overall age may affect the average age at presentation of any condition. We hypothesized that the younger overall average age in the ME is the major driver of the apparent younger age at presentation with CVDs, including AVB. As such, we conducted this analysis to report the average age at presentation with AVB in the ME and to quantify the effect of being from the ME, adjusted for the young overall population.

## Methodology

### Study design

This was a retrospective analysis of two multicenter, international prospective registries.

### Data source

Data for this study were obtained from two prospective cohort registries: PANORAMA 1 and PANORAMA 2 registries [14,15]. These registries investigated the characteristics of patients who received cardiac rhythm devices (CRM) in multiple countries worldwide, excluding North America and Australia. A standardized digital case report collected the data for both registries at study entry and at follow-up visits for a minimum of 1 year. The data included demographics, clinical features, medical history, and medications for cardiovascular conditions as baseline data. It also included the type of device and implant data. PANORAMA 1 enrolled patients from 2005 to 2011, and PANORAMA 2 from 2012 to 2016. Supplementary Table 1 shows the list of countries included in each registry. In total, 43 countries were included in this study. PANORAMA 1 included 8,586 patients, while PANORAMA 2 included 4,706 patients [14,15].

### Variables

The outcome variable of interest in this study was the average age of presentation with AVB. Independent variables tested were being from the ME and being from a ‘young country’. AVB was defined as any form of atrioventricular conduction disorder that was deemed to require pacing by the treating physician. The definition of ‘young countries’ was based on the median age of each country’s population. In order to have a consistent source of data for the definition, we used the data reported by the United Nations’ data-portal to determine the median age of the populations [13]. We observed a consistent increase in populations’ median ages between the two time periods of PANORAMA 1 and 2 [13]. Therefore, we used two median age cutoff points for ‘young country’, depending on which registry was used. We used a cutoff of ≤30 years median age in 2010 for patients from PANORAMA 1, and a cutoff of ≤35 years median age in 2015 for patients from PANORAMA 2. Supplemental Table 1 shows the median age of all involved countries and a list of all ‘young countries’, as reported by the United Nations’ data-portal [13]. Countries from the Middle East included: Egypt, Kuwait, Saudi Arabia, and Tunisia. For the purpose of this study, we only included patients who received a de novo CRM device. The study included multiple types of CRM devices, which were categorized for the purpose of this study into ‘Pacemaker’ and ‘Other devices (ICD/CRT)’. The ‘Other devices (ICD/CRT)’ variable referred to a subset of patients who had an indication for ICD/CRT on top of AVB.

### Ethical considerations

The data used in this study were de-identified, secondary, published data that did not have any direct or indirect identifiers available to the authors. Both registries used in this study were previously approved by multiple Institutional Review Board committees in all involved regions [14,15]. Both registries protocol conforms to the ethical guidelines of the 1975 Declaration of Helsinki, as reflected in a priori approval by their institution’s human research committee. Original studies were funded by Medtronic, Inc [14,15].

### Data accessibility

The data used in the study were based on secondary analysis of the Panorama registries [14,15], and the authors had no direct access to the database. For queries related to accessing the data, interested parties should contact the original registries [14,15].

### Statistical analysis

We presented continuous data as mean (±standard deviation) and categorical data as counts (proportions). Student’s t-test, Chi Square test, and Fisher’s exact test were used when appropriate to analyze data. Linear regression analysis was performed to assess the effect of being from the ME on the age at presentation with AVB, adjusted for being from a ‘young country’. Multivariate linear regression was performed, as the aim of this analysis was to estimate the mean change in the age of presentation using the regression coefficients reported in the model. This method of estimation cannot be calculated using multivariate logistic regression alone. As such, both linear and logistic regressions were performed and reported.

Analyses were performed using SAS (version 9.4; The SAS Institute, USA), and P values of <0.05 were considered statistically significant.

## Results

### Overall cohort

We included 5,259 AVB patients. Table 1 shows baseline characteristics and implant data for the overall AVB cohort. The mean age at presentation overall was 69.1 (±14.8), with 3,288/5,259 (62.5%) of patients being male. Most patients received pacemakers (4,353/5,229 [83.2%]), and a total of 1,978/5,259 (37.6%) patients were from a ‘young country’. Additional comparisons of baseline characteristics stratified by sex and region were also performed in Supplementary Tables 2 and 3, respectively. Male patients were more likely to have other co-morbidities, including diabetes [(26.5% (509/1,922) vs. 30.8% (996/3,236)], dyslipidemia [29.0% (570/1,963) vs. 37.0% (1209/3268)], and coronary artery disease [14.9% (292/1,960) vs. 33.4% (1,088/3,258), P value <0.001 for all)]. Male patients were also more likely to be smokers [8.2% (157/1910) vs. 38.0% (1220/3209), P value <0.001], and have a lower ejection fraction (56.6 ± 14.1 vs. 49.4 ± 16.3, P value <0.001). Regarding regions, the ME had the lowest mean age at presentation, while Western Europe had the highest (62.9 ± 17.8 vs. 73.5 ± 11.8, P value <0.001). Patients from ME also had the highest proportion of individuals with diabetes mellitus, while those from South Africa had the lowest [46.9% (300/639) vs. 15.3% (62/405), P value <0.001].

**Table 1:** Characteristics of the overall population.


CLINICAL CHARACTERISTICS	TOTAL (N = 5259)

**Demographics**	

Age, Mean ± SD	69.1 ± 14.8

Sex (Male)	3,288/5,259 (62.5%)

BMI (kg/m²), Mean ± SD	26.9 ± 5.1

From a ‘young country’*	1,978/5,259 (37.6%)

**Device and implant characteristics**	

Pacemaker	4,353/5,229 (83.2%)

Other devices (ICD/CRT)**	876/5,229 (16.8%)

**Single or dual chamber**	

Single-chamber	772/4,281 (18.0%)

Dual-chamber	3,509/4,281 (82.0%)

**Implant setting**	

Operating room	804/3,348 (24.0%)

EP/cardiac catheter lab	2544/3,348 (76.0%)

**Medical history**	

Hypertension	3,278/5,250 (62.4%)

Diabetes mellitus	1,505/5,158 (29.2%)

Dyslipidemia	1,779/5,231 (34.0%)

Coronary artery disease	1,380/5,218 (26.4%)

Prior myocardial infarction	702/5,234 (13.4%)

Prior CABG	427/5,257 (8.1%)

Atrial fibrillation	1,025/5,243 (19.5%)

**Smoking status**	

Never smoked	3,318/5,119 (64.8%)

Previous smoker	424/5,119 (8.3%)

Current smoker	1,377/5,119 (26.9%)

**Blood pressure**	

Systolic blood pressure, Mean ± SD	136.1 ± 23.4

Diastolic blood pressure, Mean ± SD	72.8 ± 12.9

**NYHA classification (categorical)**	

I	509/5,123 (9.9%)

II	1,090/5,123 (21.3%)

III	704/5,123 (13.7%)

IV	101/5,123 (2.0%)

**LVEF (%)**, Mean ± SD	52.0 ± 16.0

**CHADS_2_ (categorical)**	

0	805/5,126 (15.7%)

1	1,435/5,126 (28.0%)

2	1,701/5,126 (33.2%)

3	825/5,126 (16.1%)

≥4	360/5,126 (7.0%)


AVB: Atrioventricular block. SD: Standard deviation. BMI: Body mass index. CABG: Coronary artery bypass grafting. NYHA: New York Heart Association functional classification. LVEF: Left Ventricular Ejection Fraction. *‘young country’ was defined as countries with a median population age of ≤30 (PANORAMA 1) or ≤35 (PANORAMA 2), based on data from the United Nations’ data-portal. **Other devices refer to: Implantable Cardioverter Defibrillator, Cardiac Resynchronization Therapy Pacemaker, and Cardiac Resynchronization Therapy Defibrillator.

### The Middle East cohort compared to the rest of the world

Table 2 compares patients from the ME to those from other regions. There were 640/5,259 (12.16%) AVB patients from the ME. All patients from the ME were from ‘young countries’, whereas 1,338/4,619 (29.0%) of patients from other regions were from ‘young countries’. Patients from the ME had a lower mean age at presentation by seven years compared to other regions (62.9 ± 17.8 vs. 70 ± 14.1, P value <0.001). Patients from the ME were more likely to have diabetes mellitus [300/639 (46.9%) vs. 1,205/4,519 (26.7%), P value <0.001], dyslipidemia [276/639 (43.2%) vs. 1,503/4,592 (32.7%), P value <0.001], and coronary artery disease [198/638 (31.0%) vs. 1,182/4,580 (25.8%), P value = 0.005]. In addition, the left ventricular ejection fraction was significantly lower in patients from the ME as compared to those from other regions [47.5 (±16.1) vs. 52.8 (±15.8), respectively; P value <0.001].

**Table 2:** Comorbidities and risk factors of AVB in the Middle East compared to the rest of the world.


CLINICAL CHARACTERISTICS	MIDDLE EAST(N = 640)	REST OF THE WORLD(N = 4619)	P VALUE

**Demographics**			

Age, Mean ± SD	62.9 ± 17.8	70.0 ± 14.1	<0.001

Sex (Male)	373/640 (58.3%)	2,915/4,619 (63.1%)	0.018

BMI (kg/m²), Mean ± SD	28.6 ± 6.3	26.6 ± 4.8	<0.001

From a “young country”*	640/640 (100.0%)	1,338/4,619(29.0%)	<0.001

**Device and implant characteristics**			<0.001

Pacemaker	489/639 (76.5%)	3,864/4,590 (84.2%)	

Other devices (ICD/CRT)**	150/639 (23.5%)	726/4,590 (15.8%)	

**Single or dual chamber**			<0.001

Single-chamber	172/549 (31.3%)	600/3,732 (16.1%)	

Dual-chamber	377/549 (68.7%)	3,132/3,732 (83.9%)	

**Implant setting**			<0.001

Operating room	14/530 (2.6%)	790/2,818 (28.0%)	

EP/cardiac catheter lab	516/530 (97.4%)	2,028/2,818 (72.0%)	

**Medical history**			

Hypertension	395/639 (61.8%)	2,883/4,611 (62.5%)	0.729

Diabetes mellitus	300/639 (46.9%)	1,205/4,519 (26.7%)	<0.001

Dyslipidemia	276/639 (43.2%)	1,503/4,592 (32.7%)	<0.001

Coronary artery disease	198/638 (31.0%)	1,182/4,580 (25.8%)	0.005

Prior myocardial infarction	80/636 (12.6%)	622/4,598 (13.5%)	0.510

Prior CABG	63/640 (9.8%)	364/4,617 (7.9%)	0.089

Atrial fibrillation	65/640 (10.2%)	960/4,603 (20.9%)	<0.001

**Smoking status**			<0.001

Never smoked	452/638 (70.8%)	2,866/4,481 (64.0%)	

Previous smoker	56/638 (8.8%)	368/4,481 (8.2%)	

Current smoker	130/638 (20.4%)	1,247/4,481 (27.8%)	

**Blood pressure**			

Systolic blood pressure, Mean ± SD	129.9 ± 22.5	136.5 ± 23.4	0.007

Diastolic blood pressure, Mean ± SD	66.4 ± 13.1	73.2 ± 12.7	<0.001

**NYHA classification (categorical)**			<0.001

I	55/639 (8.6%)	454/4,484 (10.1%)	

II	86/639 (13.5%)	1,004/4,484(22.4%)	

III	64/639 (10.0%)	640/4,484 (14.3%)	

IV	17/639 (2.7%)	84/4,484(1.9%)	

**LVEF (%)**, Mean ± SD	47.5 ± 16.1	52.8 ± 15.8	<0.001

**CHADS_2_ (categorical)**			<0.001

0	133/638 (20.8%)	672/4,488 (15.0%)	

1	140/638 (21.9%)	1,295/4,488 (28.9%)	

2	222/638 (34.8%)	1,479/4,488 (33.0%)	

3	112/638 (17.6%)	713/4,488 (15.9%)	

≥4	31/638 (4.9%)	329/4,488 (7.3%)	


AVB: Atrioventricular block. SD: Standard deviation. BMI: Body mass index. CABG: Coronary artery bypass grafting. NYHA: New York Heart Association functional classification. LVEF: Left Ventricular Ejection Fraction. *‘young country’ was defined as countries with a median population age of ≤30 (PANORAMA 1) or ≤35 (PANORAMA 2), based on data from the United Nations’ data-portal. **Other devices refer to: Implantable Cardioverter Defibrillator, Cardiac Resynchronization Therapy Pacemaker, and Cardiac Resynchronization Therapy Defibrillator.

### Regression analysis

Table 3 shows the results of the multivariate logistic regression for the mean age at presentation. Being from a ‘young country’ and being from the ME were both independently associated with a younger age at presentation (P value 0.001). Table 4 highlights the results of multivariate linear regression: being from a ‘young country’ was associated with a mean change of –5.6 years (95% CI –6.5––4.6), whereas being from the ME was associated with a lower mean change of –3.1 years [(95% CI –4.5––1.8); P value <0.001 for both].

**Table 3:** Multivariate logistic regression for age of presentation less than 50 years.


VARIABLE	OR(95% CI)	P VALUE

**Middle East**	1.54(1.19–2.00)	0.001

**Young Country**	2.15(1.74–2.67)	<0.001


CI: Confidence interval. OR: Odds ratio.

**Table 4:** Multivariate linear regression for mean age at presentation.


VARIABLE	MEAN CHANGE(95% CI)	P VALUE

**Middle East**	–3.1(–4.5––1.8)	<0.001

**Young Country**	–5.6(–6.5––4.6)	<0.001


CI: Confidence interval.

## Discussion

In this international registry, we found a lower mean age at presentation with AVB in patients from the ME as compared with other regions. This difference was mostly due to the younger overall population in the ME; however, being from the ME was independently associated with a three-year younger age at presentation.

Our finding of a lower mean age at presentation of AVB patients in the ME is consistent with the findings of previous registries of other CVDs [2,3,4,5,6,7,8,9,10,11,12]. While there is limited literature about the characteristics of AVB patients in the ME, every other CVD studied in the ME had a similar observation [2,3,4,5,6,7,8,9,10,11,12]. Indeed, ACS registries in the ME reported the mean age at presentation to be 54–56 years, as opposed to 65–68 years in ACS registries conducted in western countries, at a similar time period [2,3,4,5,6,7,16,17,18]. Similarly, the HEart function Assessment Registry Trial in Saudi Arabia (HEARTS) and Gulf Care registries reported a mean age at presentation with heart failure to be 58 years, which is ≥10 years younger than heart failure registries in both the United States and Europe [8,9,19,20]. Lastly, atrial fibrillation registries in the ME, such as the Gulf Survey of Atrial Fibrillation Events (Gulf SAFE) registry, reported a mean age at presentation of 57 years, whereas the mean age at presentation with AF in the United States and Europe was found to be around 68 [11,21,22].

**Figure 1:**
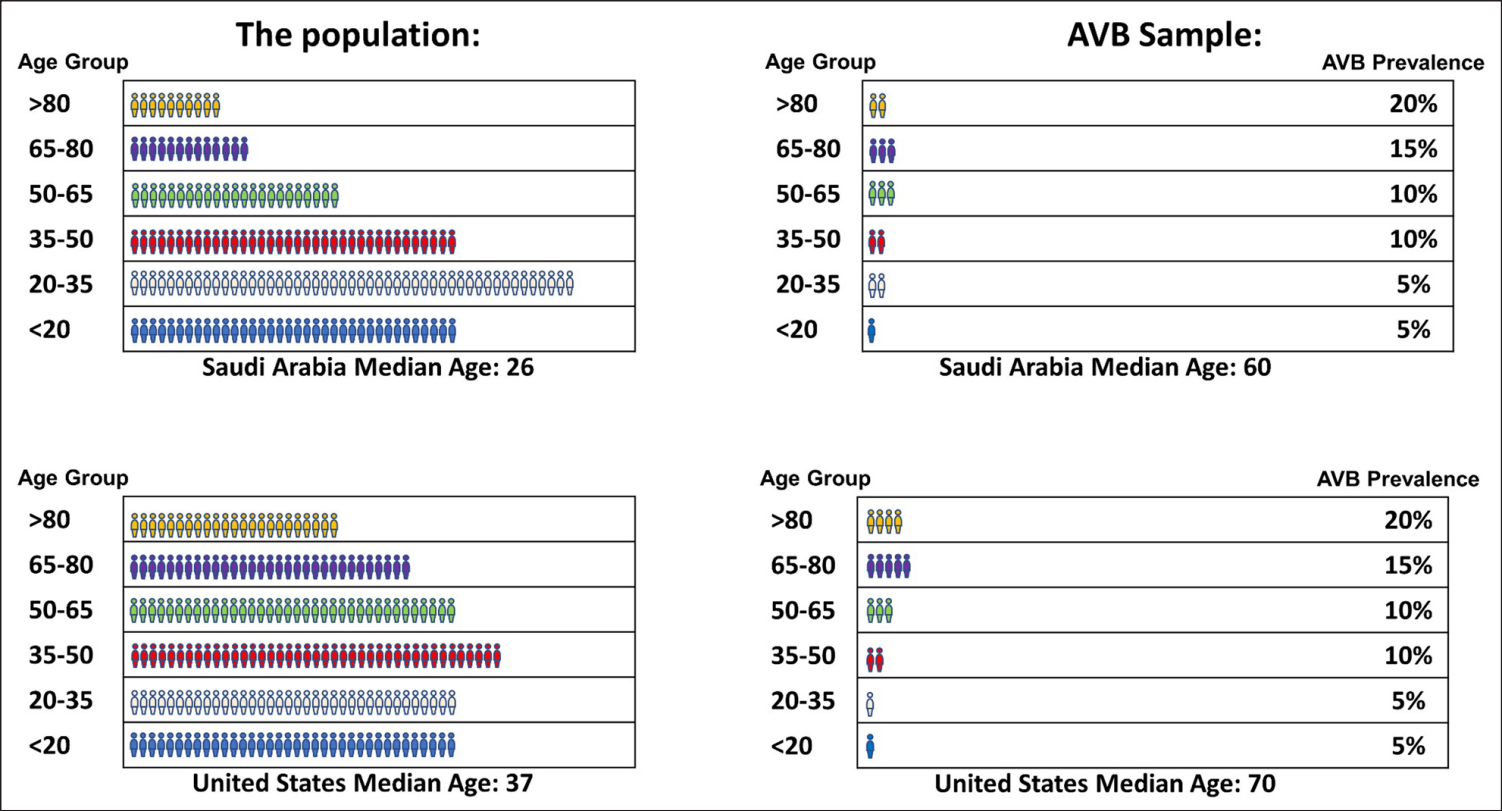
An illustration highlighting the effect of the average age of populations on lowering the average age at presentation, even if the age-adjusted prevalence is similar between the two populations. Despite a hypothetically similar prevalence of AVB in each age group, the mean age at presentation with AVB is 10 years younger due to a 10-year difference in the average age overall. AVB: atrioventricular block.

These observations have led to the notion that patients in the ME present ≥10 years earlier with CVD, due to high prevalence of risk factors such as diabetes mellitus, hypertension, and obesity [23,24]. However, simple comparison between summary statistics of registries from different regions is not accurate. One needs to adjust for differences in the age of the population in general before drawing any conclusions. Figure 1 illustrates the effect of the average age of populations on lowering the average age at presentation, even if the age-adjusted prevalence is similar between the two populations. In line with that, the average age at presentation with conditions that are not related to cardiac risk factors has also been reported to be ≥10 years younger than western countries [25,26,27,28]. For example, the average age at presentation with thyroid dysfunction and colon cancer in the ME is ≥10 years younger than the west [25,26,27,28].

Falsely assuming that patients from the ME present ≥10 years may lead to poor clinical judgments. Age is an essential determinant of the pre-test probability of many cardiac conditions, which dictates the appropriate evaluation that patients should receive. For example, guidelines recommend performing an angiogram to look for coronary artery disease (CAD) based on the pre-test probability, which is heavily dependent on the patient’s age [29]. Assuming that patients from the ME present ≥10 years earlier might lead to more invasive procedures. Also, the cause of sudden cardiac death (SCD) is largely based on the age of the deceased. This is why consensus documents recommend family screening if death occurs before the age of 50, given that CAD is the predominant cause at an older age [30]. Falsely assuming that patients from the ME present with CVD ≥10 years younger might lead to assuming that CAD is the predominant cause of SCD at a younger age and, as such, missing the opportunity to investigate other causes of SCD.

While the apparent younger age at presentation with AVB is largely due to the overall young population in the ME, being from the ME appears to be independently associated with a younger age at presentation. This, at least in part, is due to the higher prevalence of cardiac comorbidities. In our study, we found that patients from the ME had higher rates of diabetes mellitus and CAD. Previous studies have shown that both of these comorbidities are associated with earlier development of AVB [31,32,33]. There are multiple potential mechanisms that could explain this association [31,32,33]. Diabetes mellitus is associated with autonomic neuropathy and microangiopathy, leading to tissue fibrosis and disruption of the cardiac conduction system [31]. Similarly, CAD is associated with permanent fibrosis of the conduction system [33]. Kerola et al. showed in their population-based cohort study that 11% of AVB could be avoided by maintaining normal blood glucose levels [33]. An important implication of this earlier presentation is the age cutoff used to investigate secondary causes of AVB. Consensus documents recommend evaluating patients for cardiac sarcoidosis if they present with AVB at the age of 60 or less, based on studies performed in the west [1]. It is likely that the yield and, consequently, the cost effectiveness of this recommendation in the ME are different than in western countries. Studies examining the yield of advanced imaging in patients presenting with AVB in the ME are needed to determine the appropriate age cutoff.

Our study is the first to report the average age at presentation with AVB in the ME. However, it has several limitations that should be acknowledged. First, the definition and adjustment for ‘young countries’ using the median population age data from the United Nations are estimate-based and therefore may not be accurate. However, it was a useful tool to ensure the consistency of the definition and is likely a close estimate of the true median age. Additionally, the definition of AVB in the PANORAMA studies is subjective, as it is based on the decision made by the treating physician. Moreover, the type of CRM implanted was left to the treating team’s discretion. However, PANORAMA studies included experienced centers with EP services, and as such, the variability is expected to be minimal. Second, the study only included AVB patients who received CRM devices. Given that access to healthcare services may be different in different countries, this may have confounded the comparison of the prevalence of AVB reported, albeit less likely to be an important effect. Third, we cannot assume that our findings can be generalized to other CVDs. Further studies specific to each CVD are needed to ascertain the effect of being from the ME on the age at presentation. Last, we did not adjust for other variables that may influence the age at presentation with AVB, such as family history, comorbidities, and medications. This was not done as the goal of this study was to examine the effect of being from the ME in general and not the determinants of that effect.

## Conclusion

This study, using data from two large international registries, found that AVB patients from the ME had a lower mean age at presentation than their counterparts from other regions. This difference was mostly due to the younger overall population in the ME. However, being from the ME was independently associated with a three-year younger age at presentation with AVB. Our study highlights the importance of careful interpretation when comparing summary statistics of different registries and calls for further studies to identify determinants of younger age at presentation with AVB in the ME.

## Additional File

The additional file for this article can be found as follows:

10.5334/gh.1321.s1 Supplementary Tables. Tables 1 to 3.

## Author Note

Grants: The author Abdulelah H. Alsaeed received the prof. Alfagih research award for this study.
