# Supplementary material for: The Average Age of Atrioventricular Block Onset in Middle Eastern Patients with Cardiac Rhythm Devices Adjusted for the Overall Young Population: Insights from a Multicenter International Registry
Source: Glob Heart. 2024 Apr 25;19(1):40. doi: 10.5334/gh.1321 (PMC11049676; doi:10.5334/gh.1321)
Supplement: Supplementary Tables. — Tables 1 to 3. [file gh-19-1-1321-s1.pdf]

Supplemental Table 1: List of countries with median age in each registry.

PANORAMA 1 VS 2	Country	Median age*
1	ARGENTINA	30.5
1	AUSTRIA	41.9
1	BAHAMAS	29.4
1	BELARUS	38.9
1	BELGIUM	40.9
1	BRAZIL	29.2
1	COLOMBIA	27.5
1	CZECH REPUBLIC	39.6
1	DENMARK	40.6
1	DOMINICAN REPUBLIC	25
1	ECUADOR	25
1	ESPAGNA	40
1	GERMANY	44.3
1	GREECE	41.1
1	INDIA	25.1
1	KUWAIT	29.5
1	LATVIA	40.8
1	LITHUANIA	40.6
1	LUXEMBURG	38.9
1	MEXICO	26.2
1	NETHERLANDS	40.8
1	POLAND	38.1
1	PUERTO RICO	34.7
1	ROMANIA	39.4
1	RUSSIAN FEDERATION	38
1	SAUDI ARABIA	26
1	SERBIA	38.7
1	SLOVAKIA	37.3
1	SOUTH AFRICA	25
1	TURKEY	28.3
1	UNITED KINGDOM	39.5

1	URUGUAY	33.9
1	VENEZUELA	25.8
1	VIRGIN ISLANDS, U.S.	39.2
2	BANGLADESH	25.7
2	BELGIUM	40.9
2	BOSNIA AND HERZEGOVINA	40.7
2	CHINA	36.7
2	DENMARK	45.9
2	EGYPT	24.3
2	FRANCE	41.2
2	GERMANY	45.9
2	GREECE	43.4
2	INDIA	26.8
2	LITHUANIA	42.7
2	LUXEMBOURG	39.3
2	PAKISTAN	21.8
2	ROMANIA	41.3
2	RUSSIAN FEDERATION	38.6
2	SAUDI ARABIA	30
2	SLOVAKIA	39.2
2	SOUTH AFRICA	26.4
2	TAIWAN, PROVINCE OF CHINA	39.7
2	TUNISIA	31.3
2	UKRAINE	40

* Median age as reported by the United Nations' data-portal.

Supplementary Table 2: Baseline characteristics stratified by sex.

Clinical Characteristics	Total (N = 5259)	Female (N = 1971)	Male (N = 3288)	P value
**Demographics**				
Age, Mean ± SD	69.1 ± 14.8	68.8 ± 15.9	69.3 ± 14.1	0.696
BMI (kg/m²), Mean ± SD	26.9 ± 5.1	26.8 ± 5.7	26.9 ± 4.7	0.017
From a "young country" *	37.6% (1978/5259)	42.0% (828/1971)	35.0% (1150/3288)	<0.001
**Device and implant characteristics**				<0.001
Pacemaker	83.2% (4353/5229)	90.4% (1773/1961)	78.9% (2580/3268)	
Other devices (ICD/CRT) **	16.8% (876/5229)	9.6% (188/1961)	21.1% (688/3268)	
**Single or dual chamber**				0.004
Single-chamber	18.0% (772/4281)	20.2% (336/1667)	16.7% (436/2614)	
Dual-chamber	82.0% (3509/4281)	79.8% (1331/1667)	83.3% (2178/2614)	
**Implant setting**				0.257

Operating room	24.0% (804/3348)	23.0% (299/1302)	24.7% (505/2046)	
EP/cardiac catheter lab	76.0% (2544/3348)	77.0% (1003/1302)	75.3% (1541/2046)	
**Medical history**				
Hypertension	62.4% (3278/5250)	62.5% (1230/1969)	62.4% (2048/3281)	0.972
Diabetes mellitus	29.2% (1505/5158)	26.5% (509/1922)	30.8% (996/3236)	0.001
Dyslipidemia	34.0% (1779/5231)	29.0% (570/1963)	37.0% (1209/3268)	<0.001
Coronary artery disease	26.4% (1380/5218)	14.9% (292/1960)	33.4% (1088/3258)	<0.001
Prior myocardial infarction	13.4% (702/5234)	7.1% (140/1965)	17.2% (562/3269)	<0.001
Prior CABG	8.1% (427/5257)	2.8% (55/1970)	11.3% (372/3287)	<0.001
Atrial fibrillation	19.5% (1025/5243)	18.2% (358/1966)	20.4% (667/3277)	0.058
**Smoking status**				<0.001
Never smoked	64.8% (3318/5119)	88.7% (1694/1910)	50.6% (1624/3209)	
Previous smoker	8.3% (424/5119)	3.1% (59/1910)	11.4% (365/3209)	
Current smoker	26.9% (1377/5119)	8.2% (157/1910)	38.0% (1220/3209)	

**Blood pressure**				
Systolic blood pressure, Mean ± SD	136.1 ± 23.4	138.7 ± 25.0	134.7 ± 22.4	0.003
Diastolic blood pressure, Mean ± SD	72.8 ± 12.9	72.3 ± 13.0	73.0 ± 12.8	0.125
**NYHA classification (categorical)**				<0.001
I	9.9% (509/5123)	9.1% (176/1928)	10.4% (333/3195)	
II	21.3% (1090/5123)	21.0% (405/1928)	21.4% (685/3195)	
III	13.7% (704/5123)	11.2% (215/1928)	15.3% (489/3195)	
IV	2.0% (101/5123)	1.7% (33/1928)	2.1% (68/3195)	
**LVEF (%)**, Mean ± SD	52.0 ± 16.0	56.6 ± 14.1	49.4 ± 16.3	<0.001
**$CHADS_2$ (categorical)**				<0.001
0	15.7% (805/5126)	19.3% (370/1916)	13.6% (435/3210)	
1	28.0% (1435/5126)	27.4% (525/1916)	28.3% (910/3210)	
2	33.2% (1701/5126)	33.6% (643/1916)	33.0% (1058/3210)	
3	16.1% (825/5126)	14.4% (275/1916)	17.1% (550/3210)	

≥4	7.0% (360/5126)	5.4% (103/1916)	8.0% (257/3210)

AVB: Atrioventricular block. SD: Standard deviation. BMI: Body mass index. CABG: Coronary artery bypass grafting. NYHA: New York Heart Association functional classification. LVEF: Left Ventricular Ejection Fraction.

*"young country" was defined as countries with a median population age of ≤30 (PANORAMA 1) or ≤35 (PANORAMA 2), based on data from the United Nations' data-portal.

** Other devices refer to: Implantable Cardioverter Defibrillator, Cardiac Resynchronization Therapy Pacemaker, and Cardiac Resynchronization Therapy Defibrillator.

Continuous variables were compared across groups by Wilcoxon rank sum test. Categorical variables were assessed using Chi-Square test or Fisher's exact test, as appropriate.

Supplementary Table 3: Baseline characteristics stratified by region.

Clinical Characteristics	Total (N = 5259)	Western Europe (N=1483)	Eastern Europe (N=1066)	Middle East (N=640)	Asia (N=1039)	Latin America (N=615)	South Africa (N=416)	P value
**Demographics**								
Age, Mean ± SD	69.1 ± 14.8	73.5 ± 11.8	68.8 ± 14.0	62.9 ± 17.8	67.6 ± 14.0	69.8 ± 15.4	66.3 ± 17.1	<0.001
Gender (Male)	62.5% (3288/5259)	68.2% (1011/1483)	64.5% (688/1066)	58.3% (373/640)	61.8% (642/1039)	54.0% (332/615)	58.2% (242/416)	<0.001
BMI (kg/m²), Mean ± SD	26.9 ± 5.1	27.3 ± 4.6	28.0 ± 4.8	28.6 ± 6.3	24.5 ± 4.1	26.1 ± 4.3	27.8 ± 5.9	<0.001
From a “young country” *	37.6% (1978/5259)	0% (0/1483)	0.3% (3/1066)	100.0% (640/640)	48.2% (501/1039)	68.0% (418/615)	100.0% (416/416)	<0.001
**Device and implant characteristics**								<0.001
Pacemaker	83.2% (4353/5229)	78.1% (1140/1460)	83.8% (893/1066)	76.5% (489/639)	92.8% (962/1037)	91.2% (559/613)	74.9% (310/414)	

Other devices (ICD/CRT) **	16.8% (876/5229)	21.9% (320/1460)	16.2% (173/1066)	23.5% (150/639)	7.2% (75/1037)	8.8% (54/613)	25.1% (104/414)	
**Single or dual chamber**								<0.001
Single-chamber	18.0% (772/4281)	9.1% (111/1225)	11.6% (86/743)	31.3% (172/549)	17.4% (154/884)	24.2% (135/559)	35.5% (114/321)	
Dual-chamber	82.0% (3509/4281)	90.9% (1114/1225)	88.4% (657/743)	68.7% (377/549)	82.6% (730/884)	75.8% (424/559)	64.5% (207/321)	
**Implant setting**								<0.001
Operating room	24.0% (804/3348)	45.8% (281/614)	36.5% (356/975)	2.6% (14/530)	1.3% (4/299)	23.2% (137/590)	3.5% (12/340)	
EP/cardiac catheter lab	76.0% (2544/3348)	54.2% (333/614)	63.5% (619/975)	97.4% (516/530)	98.7% (295/299)	76.8% (453/590)	96.5% (328/340)	
**Medical history**								

Hypertension	62.4% (3278/5250)	62.2% (918/1477)	72.6% (774/1066)	61.8% (395/639)	58.9% (611/1037)	59.8% (368/615)	51.0% (212/416)	<0.001
Diabetes mellitus	29.2% (1505/5158)	27.1% (392/1446)	26.5% (271/1022)	46.9% (300/639)	34.4% (355/1033)	20.4% (125/613)	15.3% (62/405)	<0.001
Dyslipidemia	34.0% (1779/5231)	42.1% (622/1479)	44.9% (478/1064)	43.2% (276/639)	18.6% (189/1018)	13.7% (84/615)	31.3% (130/416)	<0.001
Coronary artery disease	26.4% (1380/5218)	26.9% (394/1464)	29.9% (318/1064)	31.0% (198/638)	31.3% (320/1022)	8.0% (49/615)	24.3% (101/415)	<0.001
Prior myocardial infarction	13.4% (702/5234)	16.7% (246/1472)	20.5% (219/1066)	12.6% (80/636)	7.5% (77/1029)	6.7% (41/615)	9.4% (39/416)	<0.001
Prior CABG	8.1% (427/5257)	10.1% (150/1481)	8.9% (95/1066)	9.8% (63/640)	5.2% (54/1039)	2.6% (16/615)	11.8% (49/416)	<0.001
Atrial fibrillation	19.5% (1025/5243)	27.7% (409/1477)	25.3% (269/1065)	10.2% (65/640)	11.7% (120/1030)	9.1% (56/615)	25.5% (106/416)	<0.001
**Smoking status**								<0.001

Never smoked	64.8% (3318/5119)	53.5% (736/1376)	63.9% (666/1043)	70.8% (452/638)	76.5% (795/1039)	61.8% (378/612)	70.8% (291/411)	
Previous smoker	8.3% (424/5119)	9.7% (134/1376)	8.8% (92/1043)	8.8% (56/638)	6.8% (71/1039)	5.7% (35/612)	8.8% (36/411)	
Current smoker	26.9% (1377/5119)	36.8% (506/1376)	27.3% (285/1043)	20.4% (130/638)	16.7% (173/1039)	32.5% (199/612)	20.4% (84/411)	
**Blood pressure**								
Systolic blood pressure, Mean ± SD	136.1 ± 23.4	135.9 ± 23.4	137.9 ± 23.6	129.9 ± 22.5	136.9 ± 23.5	N/A***	138.2 ± 23.4	0.060
Diastolic blood pressure, Mean ± SD	72.8 ± 12.9	72.4 ± 13.4	77.3 ± 11.7	66.4 ± 13.1	73.3 ± 12.0	N/A***	75.8 ± 12.9	<0.001
**NYHA classification (categorical)**								<0.001

I	9.9% (509/5123)	10.8% (148/1376)	15.0% (159/1060)	8.6% (55/639)	3.4% (35/1020)	13.7% (84/615)	6.8% (28/413)	
II	21.3% (1090/5123)	18.8% (259/1376)	36.9% (391/1060)	13.5% (86/639)	16.9% (172/1020)	20.0% (123/615)	14.3% (59/413)	
III	13.7% (704/5123)	14.5% (199/1376)	18.8% (199/1060)	10.0% (64/639)	15.4% (157/1020)	8.1% (50/615)	8.5% (35/413)	
IV	2.0% (101/5123)	1.3% (18/1376)	2.4% (25/1060)	2.7% (17/639)	1.8% (18/1020)	1.5% (9/615)	3.4% (14/413)	
**LVEF (%)**, Mean ± SD	52.0 ± 16.0	51.6 ± 16.3	48.9 ± 14.7	47.5 ± 16.1	57.4 ± 13.9	54.4 ± 17.1	52.8 ± 17.0	<0.001
**$CHADS_2$ (categorical)**								<0.001
0	15.7% (805/5126)	10.1% (145/1429)	12.8% (131/1021)	20.8% (133/638)	20.2% (206/1020)	15.7% (96/613)	23.2% (94/405)	
1	28.0% (1435/5126)	25.0% (357/1429)	27.3% (279/1021)	21.9% (140/638)	29.5% (301/1020)	34.4% (211/613)	36.3% (147/405)	

2	33.2% (1701/5126)	34.3% (490/1429)	34.3% (350/1021)	34.8% (222/638)	30.5% (311/1020)	34.7% (213/613)	28.4% (115/405)
3	16.1% (825/5126)	20.9% (299/1429)	17.7% (181/1021)	17.6% (112/638)	12.4% (126/1020)	11.9% (73/613)	8.4% (34/405)
≥4	7.0% (360/5126)	9.7% (138/1429)	7.8% (80/1021)	4.9% (31/638)	7.5% (76/1020)	3.3% (20/613)	3.7% (15/405)

AVB: Atrioventricular block. SD: Standard deviation. BMI: Body mass index. CABG: Coronary artery bypass grafting. NYHA: New York Heart Association functional classification. LVEF: Left Ventricular Ejection Fraction.

*"young country" was defined as countries with a median population age of ≤30 (PANORAMA 1) or ≤35 (PANORAMA 2), based on data from the United Nations' data-portal.

** Other devices refer to: Implantable Cardioverter Defibrillator, Cardiac Resynchronization Therapy Pacemaker, and Cardiac Resynchronization Therapy Defibrillator.

*** Blood pressure data was not available in the Latin America data.

Continuous variables were compared across groups by Kruskal-Wallis test. Categorical variables were assessed using Chi-Square test or Fisher's exact test, as appropriate.
